# Reliability and Validity of a Method for Assessment of Executive Functions in Drivers

**DOI:** 10.3390/bs10010037

**Published:** 2020-01-19

**Authors:** Rositsa Racheva, Zornitsa Totkova

**Affiliations:** Department of Psychology, Institute for Population and Human Studies, Bulgarian Academy of Sciences, Acad. G. Bonchev St., Bl. 6, Fl. 5, 1113 Sofia, Bulgaria

**Keywords:** executive functioning, validity and reliability, risky driving behavior

## Abstract

The quality of drivers’ performance is one of the crucial components related to road safety. One of the key cognitive characteristics related to the ability to drive safely are executive functions. The main goal of the presented research is to propose a new method (Trace-route task) for assessment of executive functions in drivers. The present article discusses the results of two consecutive studies. Study one aims to determine the validity and reliability of the method used and includes 134 participants, equally divided in two groups—people with disturbances in executive functions and people from the general population. Study two aims to assess the ability of the method to distinguish drivers with risky behavior. It includes 1440 participants divided in two groups—people with and without actual risky driving behavior. The results from the studies show that people with different neurological or psychiatric diseases and drivers with different road violations demonstrate worse planning ability, working memory, decision making, and cognitive flexibility. This data show that the trace-route task method is a valid and reliable instrument for assessing executive functions and has the ability to distinguish people with risky driving behavior from those who drive safely. This study reveals that the proposed method can be used for implementation in the area of traffic psychology.

## 1. Introduction

Road safety is a topic which has received rapidly growing interest over the last few decades. According to information from the World Health Organization, (WHO), more than 1.35 million people die on the roads every year. In addition, between 20 and 50 million people suffer non-fatal injuries, with many incurring a disability as a result of their injury [1]. Analyses related to accident research show that there is a variety of factors related to traffic crash causation. These factors usually are grouped into three main categories: environmental factors (e.g., limited visibility, road design, and weather), human factors (e.g., distractions, speed, and risk awareness) and vehicles related factors (e.g., blind spots, tire explosions, and technical errors). Very often these factors are interrelated (for example, slow driver reactions during bad weather conditions). However, a major factor that contributes to road accidents is human error, which is mostly connected to specific personality traits and cognitive disturbances. A large number of studies show that safe driving requires a good level of cognitive functioning [2,3,4,5,6]. One of the key cognitive characteristics related to driving is executive functions [7]. Research shows that disturbances in executive functions often result in poor driving performance. People with dementia for example perform worse on tasks measuring executive function in comparison to people from the general population [8]. Ageing also seems to be amongst the factors leading to disturbances in executive functioning and to driving the performance of people [9,10]. Adrian and colleagues present the concept that defines executive functions as processes that control and regulate thoughts and behavior [9]. Other authors describe executive functions as a construct that encompasses high-order regulatory processes of cognition [11]. Although there are a lot of definitions, it is widely accepted that the term “executive functions” is an umbrella term comprising a wide range of cognitive processes and behavioral competencies which include verbal reasoning, problem-solving, planning, sequencing, the ability to sustain attention, resistance to interference, utilization of feedback, multitasking, cognitive flexibility, the ability to deal with novelty, etc. [12]. Asimakopulos and colleagues outline six components considered to be related to driving capacity in particular: working memory; cognitive flexibility; decision making/judgement; impulse control/inhibition; self-awareness/ insight and planning. The authors pay special attention to the fact that assessment of executive functions in drivers is a serious challenge due to the fact that assessment tools usually measure only some of the components of these functions on one hand and because there is no precise combination of components of the executive functions, whose psychometric characteristics are clearly related to the results of drivers’ performance on the road on the other hand. They made a detailed analysis of the tools used to measure executive function in relation to driving and ended up with the conclusion that the Maze task (a two to five minutes long pen and paper task measuring the planning ability of the respondents) is amongst the tools with the best predictive validity in relation to driving (with a sensitivity of 78% and specificity of 82%) [11]. The method was initially developed for clinical practices [13], but later on its applicability in the assessment of fitness to drive was proven. A study of Snellgrove using this tool for assessment of executive functions clearly indicates that it discriminates with high accuracy between the participants who pass the on-road test from the ones who failed the same test [14]. Another study clearly indicates that performance on Maze tasks is predictive of prospective crashes and may be useful as a complement to other established cognitive screening tools in identifying at-risk older drivers [15]. The proposed current study tool for measurement of executive functions (Trace-route task) similarly to Maze tasks includes planning ability as a key component related to the completion of the task, but also integrates working memory, decision making, and cognitive flexibility components. It is based on Platonov’s interlaced lines and is modified especially for the purposes of assessment of executive functions in drivers. 

The main goal of the research is to propose a new method for assessment of executive functions in drivers. In order to accomplish this goal, two different studies are conducted and the results are presented in the following sections. 

## 2. Materials and Methods

This section presents information for the principles of selections and modification of the method for assessment of the executive functions in drivers, the participants included in the studies, the procedures of the research, and the statistical methods used for the analysis of the data.

The authority that provided ethical approval for conducting studies involving humans is the Internal Ethical Committee of the Institute for Population and Human Studies. The ethical approval code is: 00049.

### 2.1. Principles for Selection and Modification of the Method for Assessment of Executive Functions in Drivers

During the selection process, a number of methods for the assessment of executive functions were examined and analyzed [16,17,18,19,20,21]. The main principles for selection of an appropriate method were related to the specifics of the driving on one hand and to the specifics of the process of psychological assessment of drivers in Bulgaria on the other hand. These principles led to the selection of the following criteria that should be covered by the method used for the assessment of executive functions in drivers: a short time for implementation of the method (related to the factor “time for reaction” which influences the drivers’ performance on the road); presence of key components of executive functioning, related to driving; applicability in a group context; comparability of the participants with the normative data for certain age groups; and purposes of application for a driving license. An important factor related to both the selection and modification of the tool is the assumption that the tool for assessment of executive functioning in drivers has to be based on a higher level of difficulty than the assessment made in the clinical practice. This is important as reaction time in decision making is of a paramount importance for the process of driving. In the following paragraph, the selected method, and its modification in compliance with the presented selection criteria is briefly presented.

The method for assessment of executive functions was the Trace-route task (Modification of Platonov’s interlaced lines). Platonov’s “Interlaced lines” is a method originally used for assessment of some characteristics of attention. However, over the last few decades, methods based on “tracing” tasks have been widely used for assessment of executive functions of drivers and are considered to have a very high level of prognostic value in terms of driver performances on the road [13,14,15]. After considerable modification of the method, it has been tested for reliability and validity and further used for assessment of executive functioning of drivers. The modified tool underwent the following procedures: a change in the concept and identification of key components of executive functioning within the Trace route task, including the planning ability, working memory, decision making and cognitive flexibility; modification of the content of the method—a new interface of the tool, developed by a graphic designer with a reduced number of routes; additional modification of the content based on the observations and analyses of the researchers during the initial approbation of the tool; change of the procedure and the conditions for performing the assessment in group, for a fixed amount of time, providing the opportunity for decision making through choice of an approach for solving the task; modification of the processing and analysis of the results—only the variables that best distinguish people with cognitive impairments were taken into account in the analysis of the results. Thus, the performance of the Trace-route task encompassed most of the components of the executive functions considered to be related to driving capacity in particular.

The method used consisted of a form with 15 routes with equal length. Each of them began on one of the sides of the sheet and ended on the other side of the sheet. The routes were numerated from 1 to 15 on the left side of the sheet. The task for the respondents was to trace every route and to write down its number at the end of the route at the right side of the sheet. The task was performed for a certain amount of time (90 seconds). The instructions provided an opportunity for the respondents to make their own decisions how to deal with the task within the fixed time frame. 

In addition to the Trace-route task, a questionnaire for the collection of demographic data (in Study 1 and Study 2) and data related to risky driving behavior (in Study 2) was applied in the research.

### 2.2. Participants included in the Studies and Procedures of the Research

#### 2.2.1. Study 1

The aim of study one was to determine validity and reliability of the modified method for assessment of executive functions. Our hypothesis is that the Trace-route task will be a reliable and valid instrument that can identify people with disturbances in executive functions.

The study included 134 participants, 70 male and 64 female, between 20 and 80 years old (M = 47.11; SD = 13 = 78). Most of the people in the sample had obtained a secondary education (46.3%) or a university degree (46.3%). People with primary education (3.7%) and with doctoral degrees (3.7%) were poorly represented. Half of the participants represented the experimental group and the other half were the control group. The experimental group included patients that were identified by experts (clinical psychologists, neuropsychologists, psychiatrists, or neurologists) to have disturbances in executive functions. The respondents were receiving treatment at the time of the study. The patients were gathered by five hospitals (neurological and psychiatric) in Sofia. The control group was selected from people from the general population with similar demographic characteristics to the experimental group. 

The characteristics of the participants from the experimental group such as the demographic data (gender, age, and education) and type of disease are presented in Table 1.

The experimental group consisted of 67 participants, 35 male and 32 female, that were approximately equally distributed in the decade-based age groups. An exception is the age group “20–30 years old”, which was represented by only 4.5% of the participants. One possible explanation for this misbalance is that the patients that are hospitalized or receive day care/substitution treatment usually have serious manifestation of diseases that are not typical for the period of young adulthood. Typically the adolescence and the early adulthood are associated with the first manifestations of many diseases. Intensive treatments are rare in these periods. The educational status of the participants from experimental group is similar to those of the entire sample—most of the participants have secondary (49.2%) or high (41.8%) education. The participants from this group can be divided into four subgroups by the type of disease: patients with neurological diseases (N = 16); patients with psychiatric diseases (N = 20); patients with alcohol dependence (N = 19); and patients with drug addictions (N = 12—this sub-group consisted predominantly of patients with heroin or polysubstance addiction which were included in methadone maintenance programs). All the participants in the experimental group were previously diagnosed (by clinicians) with disturbances in the executive functioning via other tools for assessment.

The demographic characteristics (gender, age and education) of the participants from the control group are presented in Table 2.

The control group consisted of 67 participants gathered from the general population. They were identical by gender and were similar in age and education to the participants from the experimental group. 

Some of the participants were tested individually in order to observe whether they understood and adhered to the instructions given by the researcher. Other participants were tested in a group in order to check the applicability of the method in a group context. These procedures were applied both in the experimental and control groups. The observations during the testing period clearly showed that the method is applicable both in individual and in group contexts. 

The application of the tool took about three minutes (including providing instructions). For the purposes of validation of the constructed methods, the results obtained by the control and the experimental group were compared. The reliability of the methods was estimated using test-retest procedure that only involved the participants from the control group in order to establish norms for the general population. The retest was performed approximately two months after the initial testing.

#### 2.2.2. Study 2

The aim of study two was to assess the ability of the tested method to distinguish drivers with risky behavior. Our hypothesis was that people with risky driving behavior will perform worse on a Trace-route task in comparison to people without risky driving behaviors.

The study included 1440 participants from 11 Bulgarian cities. The sample consisted of an equal number of participants in all predefined age groups. The distribution of the participants by age groups is presented in Table 3.

The participants provided informed consent for their participation in the study. They were tested individually as we wanted to collect representative data for the country. The study of the executive functioning was supplemented by the collection of demographic data (age, city, etc.) as well as by data related to risky driving behavior (registered traffic violations over the last three years and suspension of the driving license). Thus the potential of the modified method to distinguish risky drivers from non-risky drivers was assessed. Both types of drivers had their registered traffic violations and driving license suspensions assessed via dichotomous questions (yes/no) and the number of “violators” was set to be equal to the number of “non-violators” for both questions in order to achieve more precise results.

### 2.3. Statistical Methods Used for the Analysis of the Data

The statistical analysis of the data from the study was performed using SPSS- version 25. The statistical methods used for the analysis of the data were Descriptive statistic; Independent Samples T-test; One-way Anova, Post Hoc Test; Pearson Correlation. 

## 3. Results

### 3.1. Results—Study 1

The results of Study 1 provide a comparative analysis of patients diagnosed with disturbances in executive functioning (experimental group) and people from the general population (control group). By using this comparison, the criterion validity of the modified method was checked. In consecutive order, results obtained via a general comparison of the control and experimental groups followed by more detailed analysis of the comparison of the control group and subgroups of the experimental group were presented. Finally, the data from the comparison of the results obtained via test-retest results of the control group is presented. Thus, the reliability of the modified method can be verified. 

In order to check the ability of the tool to distinguish people with and without disturbances in executive functioning, a comparative analysis was performed of the participants from the experimental and control group. The presence of two independent samples and the values of the scale used allowed us to apply the T-test for the purposes of the comparative analysis. The variables that distinguish the groups most are presented in Table 4.

Statistically significant differences in results of control and experimental groups on variables “Total number of answers provided by participant” (*t* = −9.864; *p* = 0.000) and “Number of correct answers provided by participant” (*t* = −10.190; *p* = 0.000) were observed. The data shows that participants from the control group managed to provide many more answers (M = 13.28) and provided many more correct answers (M = 12.87) than participants from the experimental group (Total number of answers: M = 8.52; Number of correct answers: M = 7.55). The analysis shows that the variable “number of correct answers” has a stronger ability to differentiate both groups. Therefore it was selected as a criterion for comparison of the results of the participants for future studies.

Further, more detailed comparative analysis between the data of control group and subgroups of the experimental group was conducted. The comparison of the variables that significantly distinguish the abilities of the participants related to executive functions is presented in Table 5.

The results of dispersion analysis revealed that the subgroup significantly influences the variables “Total number of answers provided by participant” (F = 24.257; *p* = 0.000) and “Number correct answers provided by participant” (F = 26.476; *p* = 0.000). Statistically significant differences can be observed between the control groups and all subgroups from the experimental group on both variables. The participants from the control group managed to provide many more answers (M = 13.28) than any of the subgroups in the experimental groups as follow: with psychiatric disease (M = 8.15), with neurologic disease (M = 9.06), with alcohol addiction (M = 8.32), and with drug addiction (M = 8.75) and to provide many more correct answers (M = 12.87) than people with psychiatric disease (M = 7.40), people with neurological disease (M = 8.56), with alcohol addiction (M = 7.21), and with drug addiction (M = 7.00). These data provide good indications of the ability of the instrument to distinguish people with different diseases with disturbances in executive functions and people from the general population. However, additional more detailed comparative studies between the participants from the general population and each of the subgroups are needed in order to provide more in-depth information on the specifics and significance of the differences in executive functions. The dispersion analysis also revealed the more discriminative qualities of the variable “Number of correct answers, in comparison to the variable “Total number of answers”, which is another reason to pick it up as a variable whose result can be a used a performance of the participants in studies of executive functions.

The reliability of the modified method was verified via a test-retest procedure. Most of the participants from the control group took part both in a test and retest stage of the study. The sample consisted of 51 of the participants from the control group that were 23–72 years-old (M = 44.35; SD = 12.69). Half of the participants have a university degree (50%), followed by those with a secondary school degree (41%). The percentage of participants with a primary school degree (2%) and a doctoral degree (3.9%) was very low. The results from the correlation analysis revealed a significant and strong relation between the performance of the participants at the test and the retest (*r* = 0.506; *p* < 0.001). These data provided sound evidence for the reliability of the modified method for assessment of executive functions.

### 3.2. Results—Study 2

The study of the ability of the method for assessment of executive functions “Trace-route task” to distinguish drivers with and without risky driving behavior revealed a significant difference between the groups when measuring “registered traffic violations in the past three years” and “suspended driving license” variables. Due to the presence of two independent samples, we applied a comparative analysis T-test. The analysis of the participants using these variables is presented in Table 6.

The comparison of the executive functions of participants with and without traffic violations in the past three years shows the presence of significant differences (*t* = −2.079; *p* = 0.038). The participants without registered traffic violations managed to trace many more routes than participants with registered traffic violations. The difference between participants with and without a suspended driving license is also significant (*t* = −2.472; *p* = 0.014). The participants whose driving licenses had been suspended manage to trace significantly less routes that those whose driving licenses had not been suspended.

## 4. Discussion

The results from Study 1 clearly show that the modified Trace-route task method demonstrates high levels of validity and reliability. Data reveal that it distinguishes people with and without disturbances in executive functions. Similarly to the results from previous studies [8], our data show that people with neurological diseases perform significantly worse on the tasks for assessment of executive functions in comparison to people from the general population. Moreover, our data shows that the tool has the potential to determine disturbances in executive functions in people with psychiatric disease or with addictions. The data suggests that patients included in the studies perform worse in terms of planning ability, working memory, decision making, and cognitive flexibility in comparison to people from the general population. However, further research with bigger sample sizes is needed to confirm the potential of the tool to distinguish disturbances in executive functioning in people with different neurological or psychiatric disorders. 

The results from Study 2 demonstrate the ability of the tool to distinguish people with and without risky driving behavior. People with registered traffic violations in the past three years and people with suspended driving license performed worse on the Trace-route task in comparison to people without traffic violations. These data confirm previous results showing the ability of Maze tasks to distinguish risky drivers [11,14]. It is clear that risky drivers demonstrate worse planning ability, working memory, decision making, and cognitive flexibility in comparison to non-risky drivers. Further, more detailed research in this direction is needed in order to specify whether other factors such as personality traits or demographic features (age, sex, etc.) are related or influence/mediate the drivers’ performance in terms of executive functions. This knowledge can be used for further improvement of interventions in the area of road safety.

The results from previous studies in the field of road safety measuring executive functions with different instruments demonstrate that executive functions can be considered as an indicator of problematic driving behavior. This fact is cross validated with other approaches for assessment of risky driving behavior [22,23]. Similarly to these findings, the results from the presented studies show that the proposed instrument is a valid tool for prevention of dangerous driving and can be used as part of different batteries for measurement of driving performance. Although the presented studies do not address the ecological validity of the instrument, this can be considered in future research with the proposed tool. 

The application of the modified tool in the process of assessment of psychological aptitude of drivers will provide valuable information both on individual and society levels. Determined disturbances in executive functions on an individual level are good points for indicative prevention (when low or moderate deviation from the norm are observed) or for referral to more detailed neurological examination (when a high deviation from the norm is observed). On a societal level, the information related to the significance of the executive functions for the process of driving can be spread through a variety of interventions among the general population as a whole (via campaigns informing people about human factors related to road safety) and drivers in particular (via general and selective prevention, via educational modules or other interventions among risky drivers, focused on importance of cognitive functioning for safe driving).

## 5. Limitations of the Study

In order to establish the potential of the instrument to distinguish people from the general population and people with different diagnoses related to disturbances in executive functions, further research in this direction is needed. More detailed comparative studies between the participants from the general population and each of the subgroups are needed in order to provide in-depth information for the specifics and significance of the differences in executive functions. In this way, specifics on disturbances of executive functions in people with different diagnoses can be found out and more precise treatment plans for these people can be proposed. 

Also further research is needed in order to check the relation of executive functions with some other factors concerning risky driving such as personality traits, demographic features, driving style, etc.

Further limitations of the studies performed concerning the research design can be addressed in the future in to improve the ecological validity of the research and the quality of the received information (based on observations from real driving situations, official recorded information about driving licenses, etc.).

## Figures and Tables

**Table 1:** Experimental group—gender, age, education and type of disease.

Gender						
Male				Female		Total
35 (52%)				32 (48%)		67 (100%)
**Age**						
20–30 years old	31–40 years old	41–50 years old	51–60 years old		Over 61 years old	Total
3 (4.5%)	16 (23.9%)	17 (25.4%)	15 (22.3%)		16 (23.9%)	67 (100%)
**Education**						
Primary	Secondary	Bachelor/Master Degree			Doctoral Degree	Total
4 (6%)	33 (49.2%)	28 (41.8%)			2 (3%)	67 (100%)
**Type of Disease**						
Psychiatric disease	Neurological disease	Alcohol addiction			Drug addiction	Total
20 (30%)	16 (23.8%)	19 (28.3%)			12 (17.9%)	67 (100%)

**Table 2:** Control group—gender, age and education.

Gender						
Male			Female			Total
35 (52%)			32(48%)			67 (100%)
**Age**						
20–30 years old	31–40 years old	41–50 years old		51–60 years old	Over 61 years old	Total
12 (17.9%)	20 (29.9%)	17 (25.4%)		9 (13.4%)	9 (13.4%)	67 (100%)
**Education**						
Primary	Secondary	Bachelor/Master Degree			Doctoral Degree	Total
1 (1.5%)	29 (43.3%)	34 (50.7%)			3 (4.5%)	67 (100%)

**Table 3:** Distribution of participants by age groups.

Age Group	Up to 25 Years Old	Between 26 and 35 Years Old	Between 36 and 45 Years Old	Between 46 and 55 Years Old	Between 56 and 65 Years Old	Over 66 Years Old	Total Number of Participants
Number of participants	240	240	240	240	240	240	1440

**Table 4:** Differences in variables that distinguish executive functions in experimental and control groups.

Variables	Group	N	M	SD	*t*	*p*
Total number of answers provided by the participant	Experimental group	67	8.52	3.466	−9.864	0.000
	Control group	67	13.28	1.897		
Number of correct answers provided by the participant	Experimental group	67	7.55	3.718	−10.190	0.000
	Control group	67	12.87	2.095		

**Table 5:** Differences in variables that distinguish executive functions in the control group and subgroups of the experimental group.

Variables		M	SD	Subgroups of the Experimental Group	M	SD	F	*p*
Total number of answers provided by participant	Control group	13.28	1.897	Psychiatry	8.15	3.675	24.257	0.000
				Neurology	9.06	3.415		
				Alcohol addictions	8.32	3.637		
				Drug addictions	8.75	3.223		
Number of correct answers provided by participant	Control group	12.87	2.095	Psychiatry	7.40	3.789	26.476	0.000
				Neurology	8.56	3.614		
				Alcohol addictions	7.21	3.980		
				Drug addictions	7.00	3.516		

**Table 6:** Comparison of the groups with and without “traffic violations in the past three years”/“suspended driving license”.

Method	Risky Behavior		N	M	SD	*t*	*p*
Trace-route task	Registered traffic violations in the past 3 years	Yes	521	11.51	2.910	−2.079	0.038
		No	521	11.88	2.812		
	Suspended driving license	Yes	310	11.53	2.925	−2.472	0.014
		No	310	12.12	2.990		

## Restrictions on the Availability of Materials or Information

The presented tool is constructed and its reliability, validity and ability to distinguish risky drivers have been proven as part of preparation of battery for assessment of drivers’ aptitude to drive requested by the Ministry of Transport, Information Technology and Information in Bulgaria. The Ministry possess the rights of the battery, therefore the authors are not allowed to fully disclose the tool in publications.
